# Tracing the impacts of recent rapid sea ice changes and the A68 megaberg on the surface freshwater balance of the Weddell and Scotia Seas

**DOI:** 10.1098/rsta.2022.0162

**Published:** 2023-06-26

**Authors:** Michael P. Meredith, E. Povl Abrahamsen, F. Alexander Haumann, Melanie J. Leng, Carol Arrowsmith, Mark Barham, Yvonne L. Firing, Brian A. King, Peter Brown, J. Alexander Brearley, Andrew J. S. Meijers, Jean-Baptiste Sallée, Camille Akhoudas, Geraint A. Tarling

**Affiliations:** ^1^ British Antarctic Survey, High Cross, Madingley Road, Cambridge CB3 0ET, UK; ^2^ Atmospheric and Oceanic Sciences Program, Princeton University, NJ 08544, USA; ^3^ National Environmental Isotope Facility, British Geological Survey, NG12 5GG, UK; ^4^ School of Biosciences, University of Nottingham, Loughborough, LE12 5RD, UK; ^5^ National Oceanography Centre, European Way, Southampton SO14 3ZH, UK; ^6^ Sorbonne Université, CNRS/IRD/MNHN, Laboratoire d'Océanographie et du Climat Expérimentations et, Approches Numériques (LOCEAN), Paris, 75005, France

**Keywords:** sea ice melt, iceberg meltwater, freshwater, isotopic tracers, Southern Ocean, Antarctica

## Abstract

The Southern Ocean upper-layer freshwater balance exerts a global climatic influence by modulating density stratification and biological productivity, and hence the exchange of heat and carbon between the atmosphere and the ocean interior. It is thus important to understand and quantify the time-varying freshwater inputs, which is challenging from measurements of salinity alone. Here we use seawater oxygen isotopes from samples collected between 2016 and 2021 along a transect spanning the Scotia and northern Weddell Seas to separate the freshwater contributions from sea ice and meteoric sources. The unprecedented retreat of sea ice in 2016 is evidenced as a strong increase in sea ice melt across the northern Weddell Sea, with surface values increasing approximately two percentage points between 2016 and 2018 and column inventories increasing approximately 1 to 2 m. Surface meteoric water concentrations exceeded 4% in early 2021 close to South Georgia due to meltwater from the A68 megaberg; smaller icebergs may influence meteoric water at other times also. Both these inputs highlight the importance of a changing cryosphere for upper-ocean freshening; potential future sea ice retreats and increases in iceberg calving would enhance the impacts of these freshwater sources on the ocean and climate.

This article is part of a discussion meeting issue ‘Heat and carbon uptake in the Southern Ocean: the state of the art and future priorities’.

## 1. Introduction

The Southern Ocean exerts a key influence on global climate, accounting for 45% to 62% of the total heat gain in the upper part of the global ocean between 2005 and 2017 [1]. It is also crucial to the partitioning of carbon between the atmosphere and the ocean, being responsible for around 40% of the total ocean uptake of anthropogenic CO_2_ [2] and for the release of old, pre-industrial CO_2_ from the deep ocean [3]. Multiple physical, biogeochemical and biological processes interact to exert these influences, including a vigorous overturning circulation [4] and strong nutrient cycling and seasonal productivity [5,6].

Freshwater injection to the surface ocean modulates these processes: at low temperatures, density gradients, and thus upper-ocean stratification, are dominated by gradients in salinity. The concentration and distribution of freshwater thus exert a strong influence on the transfer of climatically important tracers between the atmosphere and the ocean interior. The freshwater that enters the Southern Ocean derives from multiple sources. The formation and melt of Antarctic sea ice is one of the most significant seasonal signals on the planet, with impacts on light levels within the ocean (and hence phytoplankton growth and carbon fluxes), stratification and water mass production and properties. Meltwater from glaciers and ice shelves is injected at the coast and can be a source of the micronutrients that stimulate productivity [7,8]. Glacial melt can also be injected at distance from Antarctica via the calving, advection and progressive melt of icebergs [9]; these can modulate stratification locally and have the potential to enhance phytoplankton growth in their wake [10,11]. Precipitation can enter the ocean directly as snowfall or rainfall, or via accumulation on sea ice or at coastal sites with subsequent seasonal melt [12]. Changes in each of these freshwater sources have the potential to modulate the time-varying overturning circulation and properties of the Southern Ocean, with large-scale climatic consequences [13–15].

Freshwater input from these sources is changing, yielding a net Southern Ocean freshening over the past several decades [16]. Understanding the nature of the individual changes is important, since each can exert influence on the climate and ecosystem differently. The injection of glacial melt from Antarctica is increasing and is projected to accelerate in the coming decades, especially in regions of West Antarctica [1,17]. Sea ice has shown major changes in recent years: after a slow but significant circumpolar-mean increase in extent from the advent of satellite observations through to 2015, it retreated extremely rapidly during spring 2016 [18,19], with February 2022 being the record-length minimum at the time of writing. Although not well constrained by observations, high-latitude precipitation is likely to increase in a warming climate, consistent with an increase in the capacity of warmer air to hold and transport moisture [20]. The impact of icebergs has been the focus of significant attention recently, due at least partly to the gigantic (initially 5719 ± 77 km^2^) A68 iceberg that calved in 2017, and that exited the Weddell Sea and reached the shelf of South Georgia before disintegrating in 2021 [9,21].

In this paper, we use data from five occupations of an oceanographic transect spanning the Scotia and northern Weddell Seas between 2016 and 2021. During each of these cruises, samples were collected for analysis of oxygen isotopes in seawater, and we use these data alongside salinity measurements to generate new quantitative information on the time-varying upper-ocean freshwater balance in this region. Special emphasis is placed on tracing the impact of the very rapid sea ice retreat that occurred at the start of our sequence, for which the Weddell Sea was a focal point, and tracing the impact of the A68 iceberg that traversed this region toward the end.

## 2. Methods and data sources

### (a) Field area

**Figure 1.**
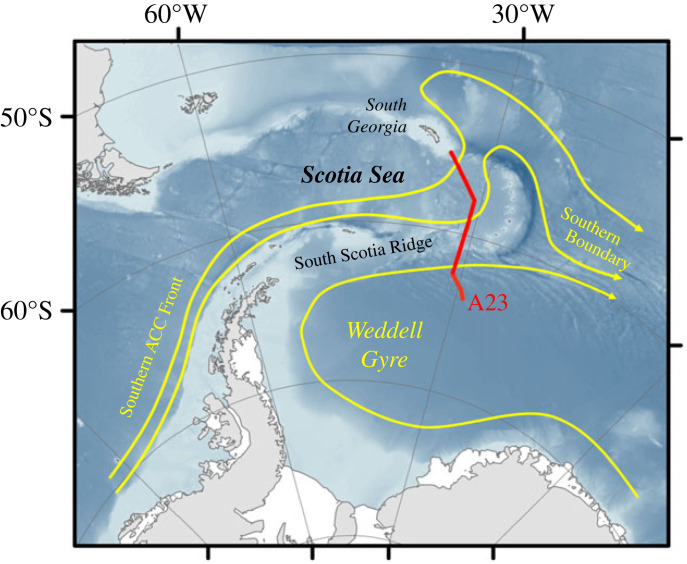
Location of the repeat hydrographic transect A23, from which data are used herein. Various topographic and oceanographic features are marked. (Online version in colour.)

Data used here were collected along the A23 repeat hydrographic section between 2016 and 2021 (figure 1; table 1). This section spans the full latitude range of the eastern Scotia Sea between South Georgia and the South Scotia Ridge, and penetrates into the northern Weddell Sea, with its southernmost extent limited by sea ice or logistical constraints (figure 1). Hydrographic data from this section have been used for many purposes previously, including tracking the circulation and progressive diminution of Antarctic Bottom Water (AABW) export from the Weddell Sea (and recent variability therein), and determining the causes of remarkably stratified abyssal waters in the Scotia Sea [22–24]. Sections used here were occupied as part of the projects ‘Ocean Regulation of Climate via Heat and Carbon Sequestration and Transports' (ORCHESTRA) and ‘ENCORE is the National Capability ORCHESTRA Extension’ (ENCORE). Pre-ORCHESTRA occupations of this transect include little or no isotope tracer sampling and were not found to contribute to the present study. A gap at the northern end of the A23 section exists in 2017; fortunately, we collected some surface samples here during the Antarctic Circumnavigation Expedition (ACE) in the same season, effectively filling the data gap for surface waters. In addition to stations along A23, we use some ancillary stations from the 2021 JC211 cruise (table 1) located close to the northern end of the section and targeted at sampling in proximity to the remnants of the A68 megaberg that lay very close to A23 at that time.

**Table 1.** Details of the cruises from which data are used here.

cruise	ship	project	occupied	principal scientist
JR15006	RRS James Clark Ross	ORCHESTRA	March–April 2016	Andrew Meijers
JR16004	RRS James Clark Ross	ORCHESTRA	February–March 2017	Jean-Baptiste Sallée
ACE	Akademik Tryoshnikov	ACE	March 2017	David Walton
JR17003	RRS James Clark Ross	ORCHESTRA	January–February 2018	Povl Abrahamsen
DY113	RRS Discovery	ORCHESTRA	February–March 2020	Yvonne Firing
JC211	RRS James Cook	ENCORE	February–March 2021	Povl Abrahamsen

The A23 section is fortuitously positioned for studies of Southern Ocean freshwater changes. Within the Scotia Sea, it spans the southernmost front of the Antarctic Circumpolar Current (the Southern ACC Front, SACCF, which loops anticyclonically around South Georgia from the south) and also the Southern Boundary of the ACC (figure 1). Farther south, the section crosses the northern limb of the Weddell Gyre, a region of predominant sea ice melt sitting downstream in the cyclonic circulation from the major ice production sites in the southern Weddell Sea [25]. Separating these two regimes is the Weddell-Scotia Confluence, which lies above and close to the topography of the South Scotia Ridge (figure 1) and which features waters of unusually weak mid-depth vertical stratification [26].

### (b) Data and sample collection

A SeaBird Scientific SBE 911plus Conductivity–Temperature–Depth (CTD) system was used on each of the ORCHESTRA/ENCORE cruises to obtain near full-depth profiles of temperature, salinity and other ocean variables. In each case, the CTD was mounted on a frame that also carried Niskin bottles; these were used to obtain discrete water samples for conductivity calibration of the CTD and the oxygen isotope samples. The exception to this modus operandi is the ACE cruise, for which only discrete near-surface samples of isotopes [27] and salinity [28] are used here, obtained from the ship's underway pumped water supply. ACE salinity samples were processed after the cruise on an OPTIMARE Precision Salinometer at the Alfred Wegener Institute, Germany. The isotope samples were collected into 50 ml glass bottles that were then sealed with a rubber stopper and aluminium crimp seal, or into 30 ml Nalgene HDPE bottles. All isotope samples were transported via dark cool stow to the British Geological Survey, where they were analysed for their oxygen isotope composition (*δ*^18^O, the standardized ratio of H_2_^18^O to H_2_^16^O in seawater), using the CO_2_ equilibration method with an Isoprime 100 mass spectrometer plus Aquaprep device. Isotope measurements were calibrated against internal and international standards including VSMOW2 and VSLAP2. The long-term mean of all seawater replicates is better than 0.04‰ (1 s.d.).

Quality control was conducted on the *δ*^18^O data, with coherence of profiles inspected and obvious fliers repeated or flagged as bad. While samples were all analysed in the same laboratory, the possibility of small offsets between cruises cannot be excluded: while factors such as the use of intermediate standards should give zero or very small changes, there is some evidence that they cannot be discounted *a priori* [29]. To account for this, we derive the mean *δ*^18^O of AABW on each section and use this to offset each cruise dataset so they have the same AABW means. (AABW is preferred to Circumpolar Deep Water (CDW) for this, despite being more recently formed at this location, since it is more voluminous and hence a more reliable mean can be obtained). Offset values are JR15006 (+0.00‰ by definition, taken to be baseline reference), JR16004 (–0.03‰), JR17003 (–0.07‰), DY113 (–0.03‰) and JC211 (+0.01‰). The offsets used are typically very close to the precision of the data. Application of these adjustments ensures the results presented here are internally consistent, and the year-on-year changes identified are not data artefacts. It is accepted that this process will include any small natural variability in deep water properties as well as any methodologically-induced changes, and thus the upper-layer changes that we derive might be marginally smaller than otherwise. However, it is the safest approach.

The sampling strategy on cruises was to enhance the vertical resolution of sample collection in the upper ocean, where gradients in overall freshwater and its constituent components are expected to be largest, and also enhance sampling density close to the seabed where bottom boundary layers can be important. Depending on water depth, typically between 8 and 20 samples were collected per cast, with nominally 6 of these being collected in the upper 200 m.

### (c) Utility of *δ*^18^O as a freshwater tracer and quantification of source water fractions

Like salinity, *δ*^18^O is set at the surface by freshwater exchanges with the atmosphere and cryosphere, and is a conservative tracer in the ocean interior. *δ*^18^O, when measured in addition to salinity, provides an extra degree of freedom that informs on the source of the freshwater inputs. On a planetary scale, the lighter H_2_^16^O molecule evaporates preferentially in the tropical/subtropical regions, while the heavier H_2_^18^O molecule rains out preferentially from the atmosphere. With a net movement of water vapour in the atmosphere towards the poles, this results in high-latitude precipitation having very low values of *δ*^18^O. Glacial and iceberg melt, which derives from this precipitation, has correspondingly very low values. Conversely, sea ice has a much higher value of *δ*^18^O, very close to the value of the seawater from which it formed (fractionation factor around 1.0026 to 1.0035 [30,31]). Consequently, concurrent measurements of salinity and *δ*^18^O can inform on the relative importance of sea ice melt and meteoric water (precipitation and glacial melt) injection to the seawater sampled.

Quantitative information on different freshwater sources can be derived via implementation of a simple three-endmember mass balance. This approach was originally developed for the Arctic [32] and has subsequently been adapted for freshwater studies in the Southern Ocean (e.g. [33]). It assumes that each water sample is comprised of three constituent components, namely sea ice melt, meteoric water and an ambient saline oceanic endmember. Here we take the oceanic endmember to be CDW, the most saline water mass along the section and the source water mass that enters the Southern Ocean from lower latitudes and from which all returning water masses are ultimately formed. The mass balance enables the separate contributions to be determined via

2.1 $$\begin{array}{rl} & {\text{F}}_{\text{sim}}+{\text{F}}_{\text{met}}+{\text{F}}_{\text{cdw}}=\text{1}\\ & {\text{S}}_{\text{sim}}\text{.}{\text{F}}_{\text{sim}}+{\text{S}}_{\text{met}}\text{.}{\text{F}}_{\text{met}}+{\text{S}}_{\text{cdw}}\text{.}{\text{F}}_{\text{cdw}}=\text{S}\\ & \delta_{\text{sim}}\text{.}{\text{F}}_{\text{sim}}+\delta_{\text{met}}\text{.}{\text{F}}_{\text{met}}+\delta_{\text{cdw}}\text{.}{\text{F}}_{\text{cdw}}=\delta \end{array}$$

where F_sim_, F_met_ and F_cdw_ are the fractions of sea ice melt, meteoric water and CDW respectively; S_sim_, S_met_ and S_cdw_ are the salinities of the pure endmembers of the respective constituent waters; *δ*_sim_, *δ*_met_ and *δ*_cdw_ are corresponding *δ*^18^O values for these endmembers; and S*, δ* are the measured salinity and *δ*^18^O of the water sample.

**Figure 2.**
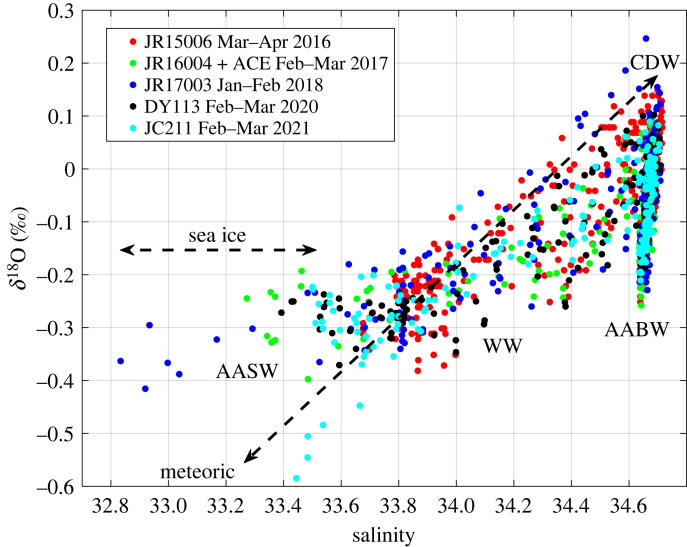
Data from all depths of the repeat occupations of the A23 section in salinity-δ^18^O space. Water masses marked are CDW, AABW, WW and AASW. Data points are coloured according to cruise (see legend). (Online version in colour.)

The values for the endmembers are given in table 2. CDW salinity and *δ*^18^O were taken from direct measurements on the cruises, and are representative of values typically encountered on the A23 section (e.g. figure 2). Other values used are as per [33], with the meteoric water endmember combining an approximate glacial input signal around the Weddell Gyre and a precipitation signal from the gyre's northern extent [34–36]. Choice of endmember values is inevitably somewhat arbitrary given the spread of values along the section and more broadly, but has negligible impact on the year-to-year changes in freshwater fraction that are the focus of this paper.

**Table 2.** Values used for the three-endmember mass balance calculations (equation (2.1)).

	sea ice melt	meteoric water	CDW
salinity	5.0	0.0	34.7
*δ*^18^O	+1.8 ‰	−18 ‰	+0.1 ‰

In previous implementations of mass balance equations for the Weddell Sea, we have adopted also a four-endmember system to reflect also the presence of Winter Water (WW), the temperature-minimum remnant of the winter mixed layer that persists into summer [33]. We do not apply this approach here because it requires an additional tracer, e.g. a hybrid tracer created from nutrients and dissolved oxygen data: this requires measurements not obtained on all the cruises used here, and such hybrid tracers are non-conservative in the shallow waters on which this paper focuses.

To quantify the impact of uncertainty in endmember selection and measurement uncertainty on freshwater percentage calculation, we conducted a sensitivity analysis as per [33]. For this, endmembers were varied according to their estimation and measurement uncertainty, and both individual and combined effects on freshwater contributions and final quantifications were assessed. Sea ice melt and meteoric water quantifications were found to be most sensitive to the choice of *δ*^18^O endmembers, and least sensitive to salinity, and the choice of meteoric water endmember was most influential overall. In total, uncertainties in the derived freshwater contributions were calculated as being less than ±1 percentage points of the water sampled.

It should be noted that the system of equations used to derive the freshwater percentages can yield negative values for sea ice melt in the area sampled. This is consistent with the addition/removal of freshwater from the ocean by sea ice being a bidirectional process, with positive values denoting net sea ice melt having occurred into the waters sampled, and negative values denoting net sea ice production from the waters sampled. For meteoric water, negative values are not expected in the high-latitude Southern Ocean, with only a few exceptions (e.g. under the influence of strong evaporation driven by intense katabatic winds or cold air outbreaks).

Here we interpret the changes in freshwater fractions between cruises as primarily reflecting interannual variability, especially for the sea ice melt component. The cruises were all conducted in the austral summer season; however, there is the possibility of some aliasing of the seasonal signal, given the different months of occupation of the section (table 1). To address this, we used a gridded seasonal salinity climatology (2005–2021) derived from Argo float data [37,38] to adjust the salinity values to account for the different seasonal timings. This approach was found to make no difference to the results presented herein, giving confidence that the results are not impacted by aliasing. In the absence of reliable year-round *δ*^18^O data, we cannot address seasonal aliasing of that signal directly; however, *δ*^18^O is relatively insensitive to sea ice melt/formation processes hence any seasonal changes are not expected to impact strongly on derived sea ice melt variability.

### (d) Cryospheric data

Interpretation of the cruise data is aided by cryospheric data from several sources. Sea ice concentration information is obtained from the Hadley Centre Sea Ice and Sea Surface Temperature (HadISST) dataset, available at https://www.metoffice.gov.uk/hadobs/hadisst/ with updates. Iceberg trajectory data is obtained from the Brigham-Young University (BYU) database [39], being a consolidated dataset comprising all available satellite scatterometer instruments plus measurements from the National Ice Center; this product is available at https://www.scp.byu.edu/data/iceberg/database1.html. Iceberg outlines were traced from MODIS Terra images downloaded from NASA's Worldview Snapshots application (https://wvs.earthdata.nasa.gov), part of the Earth Observing System Data and Information System (EOSDIS).

## 3. Changes in surface salinity and *δ*^18^O

When viewed in salinity-δ^18^O space, the role of different freshwater components in determining the water mass characteristics in the Weddell and Scotia Seas is apparent (figure 2). In this space, the input of meteoric water (in the form of precipitation, glacial melt or iceberg melt) moves the locus of points diagonally downward to the left. Conversely, reduction in meteoric water via greater mixing with CDW moves the locus of points diagonally upward to the right. Sea ice melt moves the locus of points almost horizontally to the left (fresher), while sea ice production moves the locus almost horizontally to the right (more saline).

CDW is the most saline and highest-δ^18^O water mass, being derived from the products of deep convection in the North Atlantic and representing the oceanic source from which other Southern Ocean water masses derive. AABW is slightly fresher than CDW but has significantly lower *δ*^18^O values, reflecting the net input of glacial melt in the AABW formation regions, with net sea ice production there almost completely counteracting the salinity decrease that this would otherwise represent. Above CDW lies WW, the subsurface remnant of the previous winter's deep mixed layer; this lies strongly along a diagonal mixing line from CDW, reflecting the role that meteoric water inputs play in determining salinity stratification between these water masses.

Above WW lies Antarctic Surface Water (AASW), the most variable water mass in both salinity and *δ*^18^O. Two marked clusters of AASW are immediately apparent in figure 2, namely JC211 data (cyan data points; early 2021) and JR17003 data (dark blue data points; early 2018). These clusters contrast strongly in salinity-δ^18^O characteristics: relative to the general WW properties, JC211 lies close to the diagonal meteoric water mixing line, while the locus of points for JR17003 is offset horizontally from this line. It should be noted that the AASW in the year prior to JR17003 (JR16004; early 2017; green data points) is also offset horizontally, albeit by less than JR17003. These two clusters are the primary foci of this paper.

**Figure 3.**
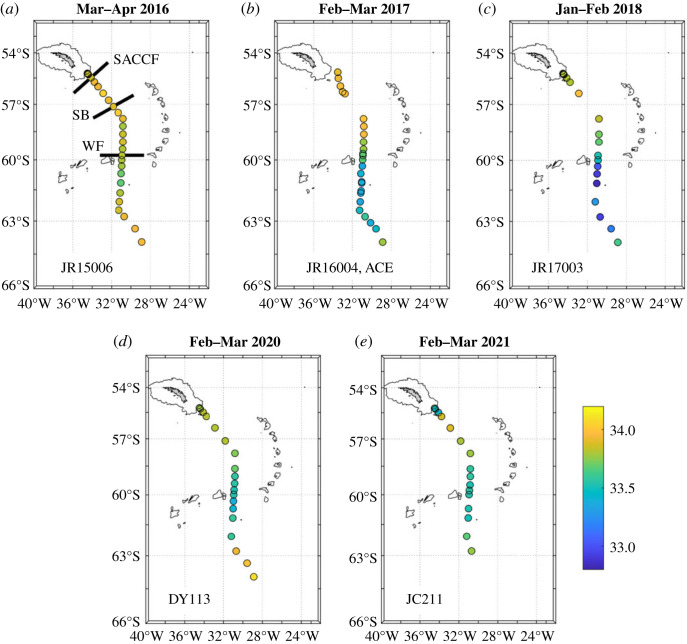
Near-surface salinity for each of the cruises used here. Data are plotted from the uppermost bottle of each cast that has a bottle in the upper 20 m. Land is shaded and the 1000 m bathymetric contour is marked. Indicative positions for the SACCF, Southern Boundary of the ACC (SB) and Weddell Front (WF) are marked in (*a*). Of particular note is the progression of upper-layer salinity in the northern Weddell Sea, which began with relatively saline waters in 2016, then freshens through 2017 to minimum values in 2018, then recovers somewhat toward higher salinities. (Online version in colour.)

**Figure 4.**
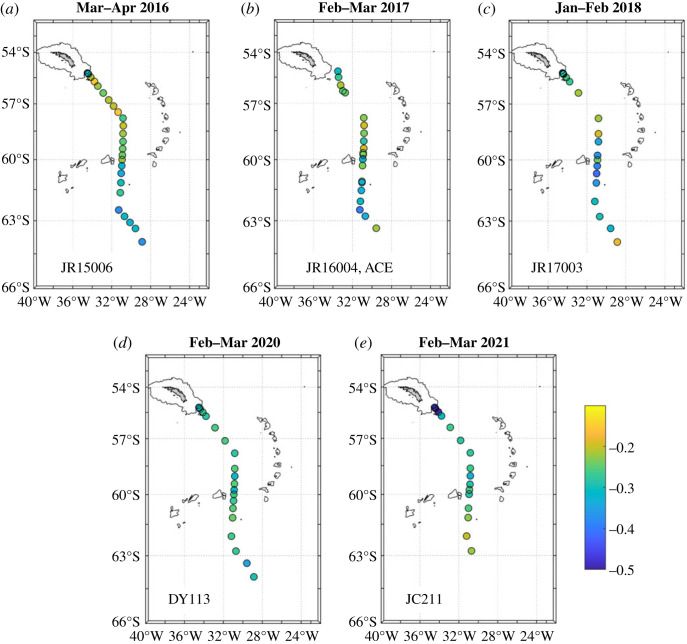
Surface *δ*^18^O for each of the cruises used here. Data are plotted from the uppermost bottle of each cast that had a sample in the upper 20 m. Land is shaded and the 1000 m bathymetric contour is marked. Note in particular the cluster of very low values immediately adjacent to South Georgia in the 2021 occupation of the section (*e*). (Online version in colour.)

The two distinct clusters also show strongly contrasting spatial distributions. Near-surface salinity along the cruise track shows a strong freshening in the northern Weddell Sea from 2016 to 2018 (figure 3*a–c*), then a return to surface salinities closer to those originally seen at the start of the sequence. During this period, the near-surface *δ*^18^O in the northern Weddell Sea showed only minimal changes (figure 4), consistent with the locus of points being near-horizontal in salinity-δ^18^O space (figure 2). The largest anomalies in near-surface *δ*^18^O occur in a small region at the northern end of the Scotia Sea adjacent to the island of South Georgia in early 2021 (figure 4*e*).

## 4. Sea ice melt distribution and changes

**Figure 5.**
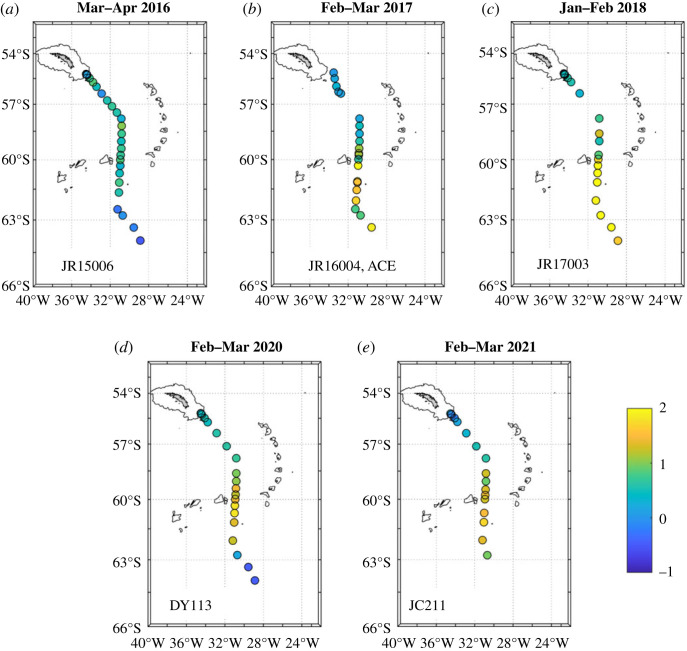
Percentage sea ice melt in the near-surface layers of the northern Weddell and Scotia Seas, derived from salinity and *δ*^18^O. Values are derived using data from the uppermost bottle of each cast that had a sample in the upper 20 m. Land is shaded and the 1000 m bathymetric contour is marked. Note in particular the strong increase in sea ice melt in the northern Weddell Sea during 2016 to 2018, and a decline thereafter. (Online version in colour.)

The patterns of salinity and *δ*^18^O anomalies are indicative of their origins, with the multi-year changes in the northern Weddell Sea being driven primarily by changes in the sea ice field, and the anomalies close to South Georgia in 2021 being caused by meteoric water input. To quantify these inputs, we implement the mass balance system given in equation (2.1). For sea ice melt in the northern Weddell Sea, the near-surface values in 2016 are as low as –1%, indicating that there had been some freshwater removal through net sea ice production from this water prior to it being sampled (figure 5*a*). During the next 2 years, the sea ice melt percentage increased strongly in the near-surface layers, reaching a maximum that exceeds 2% in early 2018 (figures 5*b,c*). There was then a decrease closer to previous values in the southernmost region, the exact timing of which is difficult to ascertain given the 2-year gap between JR17003 and DY113.

That there are some initially negative near-surface values of sea ice melt in the northern Weddell Sea (figure 5*a*) could be seen as counterintuitive, given that this is a region where typically one would expect net sea ice melt, with strong ice production instead occuring further south and over the shelves of the Weddell Sea in particular [13]. It seems likely, however, that waters from the sea ice formation regions will have advected around the Weddell Gyre to reach sampling sites on the A23 section, and that their negative values will have been exacerbated by the overall pattern of decadal sea ice change in the Weddell Sea. Sea ice here reached its record-length maximum in 2013–2015, immediately prior to the commencement of our sampling sequence [19]; it is inevitable that this anomalously extensive sea ice will have left an imprint on the ocean water from which it formed.

**Figure 6.**
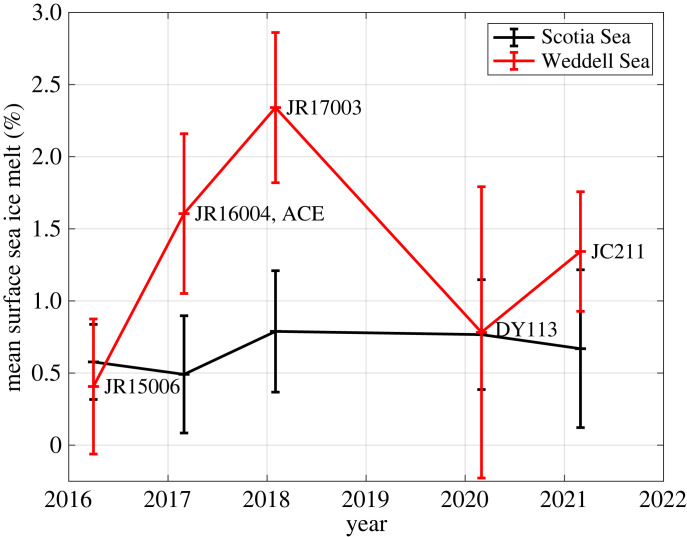
Spatial means of near-surface sea ice melt in the Scotia Sea (black) and northern Weddell Sea (red), with individual cruises marked. The division between these basins is here taken to be 60°S, and horizontal averaging conducted north and south of this latitude. (Online version in colour.)

**Figure 7.**
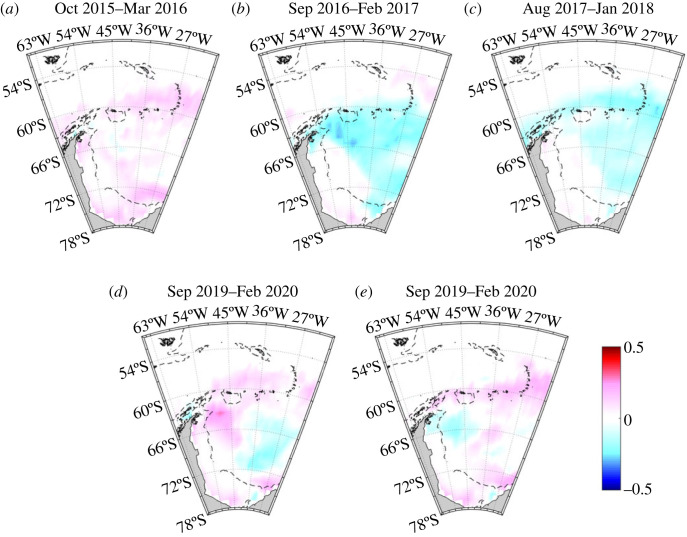
Average sea ice concentration anomalies (relative to 1979–2021) for the six months preceding each of the cruises used here. Note in particular the strong positive anomalies in the region during late 2015–early 2016, after which the anomalies became strongly negative across this part of the Weddell Sea for greater than 2 years. This is the regional manifestation of the strong sea ice retreat, during which sea ice extent collapsed from its record-length maximum in 2013–2015 to a new minimum in 2016. (Online version in colour.)

The different temporal changes in mean near-surface sea ice melt in the Scotia and northern Weddell Seas are marked. If averaged horizontally north and south of a division at 60°S, the relative invariance in the Scotia Sea is clear (black line in figure 6), with mean sea ice melt varying by less than 0.4 percentage points. By contrast, the mean sea ice melt in the northern Weddell Sea increases by approximately 2 percentage points from a minimum in 2016 to a peak in early 2018 and declines subsequently (red line in figure 6). The overall range in mean near-surface sea ice melt in the northern Weddell Sea is approximately 2 percentage points, more than five times that in the Scotia Sea. The timing of the changes in the northern Weddell Sea strongly reflects the timing of changes in the sea ice field, which showed an unprecedented retreat in spring 2016 ([19]; see also figure 7*a,b*). There is a temporal lag between the peaks of the signals, with the maximal oceanic imprint of the sea ice change occurring 1–2 years after the rapid retreat (figure 6). Part of this signal is likely to be caused by advection and spreading of the sea ice melt imprint on the ocean, with signals imparted further south in the Weddell Gyre taking some time to reach the sampling site. It is also likely that sea ice melt anomalies accumulated in the ocean for some time after the initial rapid retreat, with sea ice concentrations remaining anonalously low after this time (figure 7*b*,*c*). After the 2-year gap in our sampling, sea ice in the region was a mixed pattern (figure 7*d*,*e*); the ocean anomalies reflect this, with varied patterns of positive and negative sea ice melt along the A23 line (figure 5*d*,*e*) and intermediate mean sea ice melt values in the northern Weddell Sea (figure 6).

An important issue relates to the fate of the sea ice melt anomalies and the role of upper-ocean processes in influencing this signal. Vertical mixing of the anomalies is expected, especially during convective overturn of the upper ocean in autumn and winter. This process is likely to distribute the anomalies over a vertical range and is key to structuring the impact of these freshwater changes on upper-layer stratification, and hence exchange of climatically important tracers between the atmosphere and the ocean interior. In this context, it should be noted that sea ice production occurs generally toward the southern parts of the Weddell Sea, while sea ice melt signals predominate in the northern part [13,40]; this overall pattern of ice divergence away from the coast means that sea ice melt signals mixed down from the surface will not necessarily be simply compensated by ice production signals at depth. It is thus possible that significant amounts of sea ice melt will accumulate in the upper ocean below the surface layer.

**Figure 8.**
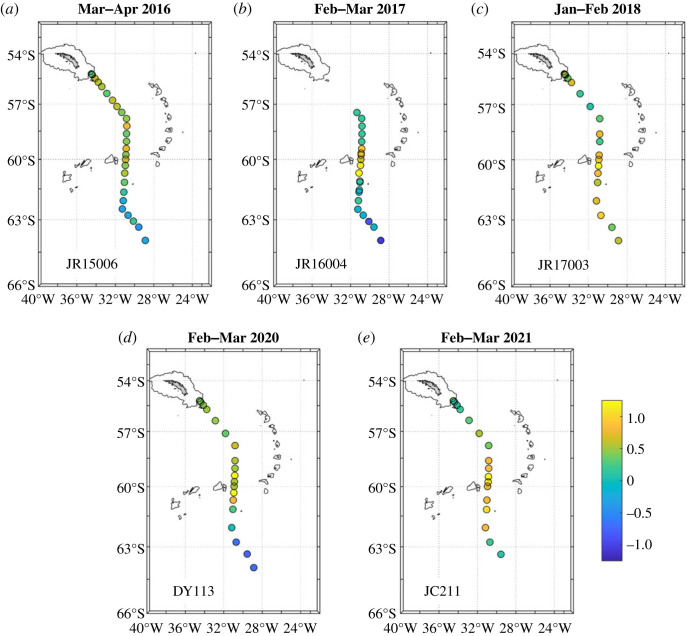
Column inventories of sea ice melt (in m), integrated over the upper 100 m. Note that ACE stations are excluded since these were surface samples only, hence inventories are not possible. Negative values indicate net freshwater removal through sea ice production. (Online version in colour.)

To gain quantitative information on the amounts of sea ice melt accumulated in the upper ocean during the unprecedented retreat, we calculate column inventories by integrating the sea ice melt percentages over the upper 100 m of the ocean. It should be noted that, while the absolute values for column integrals are sensitive to the choice of this thickness, the year-on-year changes in those values are not. By this measure, the change in height of freshwater derived from sea ice melt present in the northern Weddell Sea between early 2016 and early 2018 is approximately 1 to 2 m (figure 8*a*–*c*). Interpreting this value requires caution: it is clearly related to the excess amount of anomalous sea ice melt that entered the ocean between these times, but it does not directly reflect the mean thickness of sea ice that melted. First, it should be noted that water is denser than ice, so there is a simple scaling (approximately 0.9) that leads to the ocean tracer value underestimating the thickness of solid sea ice that melted. Second, once the melt enters the ocean it mixes laterally, reducing the peak values. Third, the isotope tracer used here detects the impact of sea ice formed from the freezing of seawater: any snow accumulation that becomes compacted on top of the sea ice will not be included in this sea ice melt calculation. The third of these factors appears to be of comparatively low significance, given the relative invariance of *δ*^18^O in the northern Weddell Sea waters that showed the large changes in sea ice melt. The other two factors both reduce the ocean signal relative to the solid sea ice change, and thus the quantification should be interpreted as an approximate lower limit to the net sea ice thickness change associated with the rapid sea ice retreat.

## 5. Meteoric water distribution and changes

**Figure 9.**
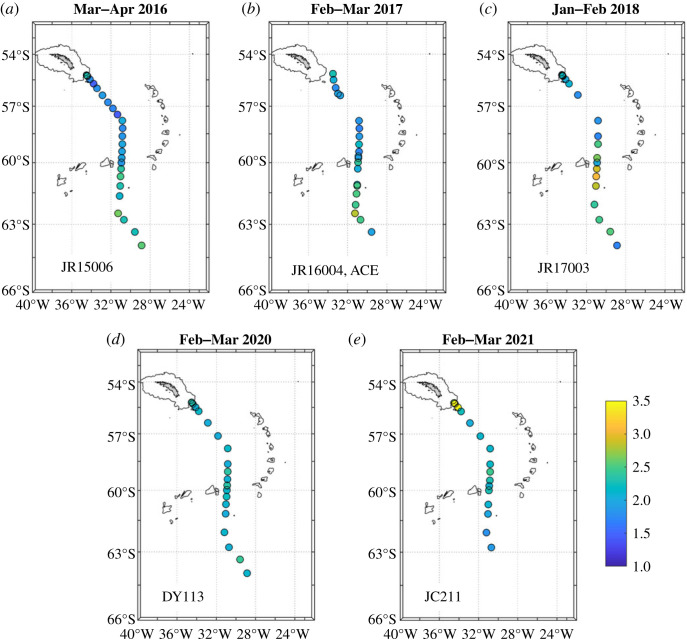
Percentage meteoric water at the surface from the cruises. Note in particular the relative invariance across the years, with the exception of 2021 very close to South Georgia (*e*). This was the time that the remnants of giant iceberg A68 were in the vicinity (figure 10), adding glacial melt to the ocean during its demise; this is seen in more detail in figure 11. Also of note are the elevated values above the South Scotia Ridge in early 2018. (Online version in colour.)

The other freshwater component that is quantified in the endmember mass balance equation is meteoric water, being the net input to the ocean from precipitation and glacial sources. Meteoric water is relatively invariant across the field area and across the years of sampling (figure 9), as expected given the relative invariance of *δ*^18^O. The major exception is adjacent to South Georgia in early 2021 (figure 9*e*), which shows a small region with meteoric water values above 3%, significantly higher than the 1–2% background values seen more broadly and at other times.

**Figure 10.**
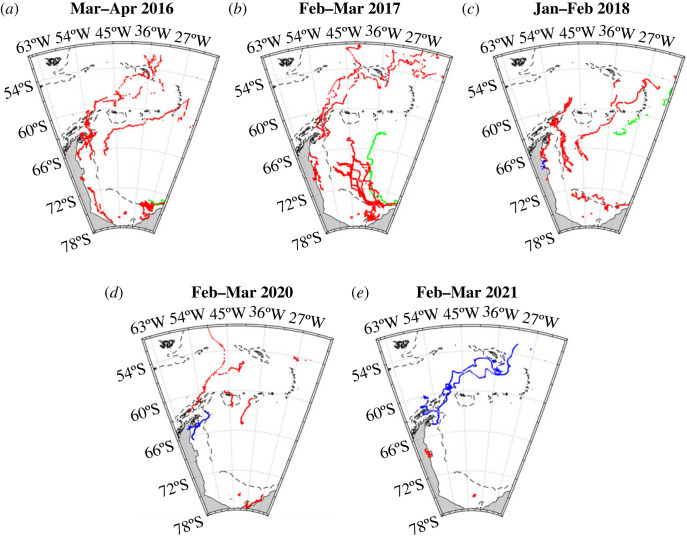
Trajectories of icebergs tracked in the Brigham Young University database. Each panel represents the positions of the icebergs at the times of the cruises used here; the coloured trajectories are 1 year in length. Blue denotes the giant A68 iceberg, which calved in the western Weddell Sea and advected across the Scotia Sea toward South Georgia before disintegrating. Green denotes iceberg A61, which was one of a cluster of icebergs that exited the Weddell Sea in 2017–2018, including crossing the A23 section. (Online version in colour.)

**Figure 11.**
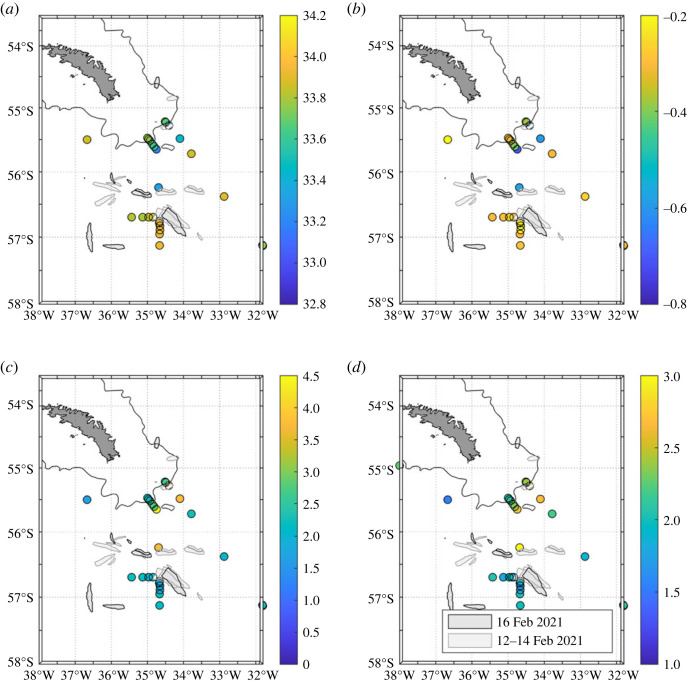
Surface values of (*a*) salinity, (*b*) *δ*^18^O, and (*c*) meteoric water, for JC211 stations in the vicinity of the giant A68 iceberg. (*d*) Column inventories of meteoric water down to 100 m depth. At the time of these stations, A68 had begun disintegrating into multiple pieces; the outlines show the size and positions of these pieces on 12–14 February and 16 February 2021. In addition to the routine A23 stations (diagonal line extending southeast from South Georgia), several other stations were occupied specifically to detect the imprint of the iceberg on the ocean. (Online version in colour.)

The source of this anomaly is meltwater from the giant iceberg A68, which originated in the Weddell Sea and traversed the Scotia Sea in the period preceding the timing of the 2021 A23 cruise (blue in figure 10*d*,*e*). By the time A68 had reached the vicinity of South Georgia, it had lost considerable mass [9] and had begun disintegrating into multiple pieces (figure 11). In addition to the routine A23 stations, extra CTD stations were occupied on cruise JC211 in the vicinity of these iceberg fragments, with the purpose of tracing and quantifying meltwater injection to the ocean. These stations showed a remarkable level of small-scale structure in the data, with many of the salinity and *δ*^18^O data points very close to iceberg fragments showing very little difference from values that would typically be expected along A23 in other years (figure 11*a*,*b*). In particular, short zonal and meridional sections to the west and south of the largest iceberg fragment show salinities of 33.8–34.0 and *δ*^18^O values around –0.3‰, not dissimilar to the general values for this location at other times (figure 2). Conversely, some markedly different values exist to the north of this large fragment, clipping the end of a very short section that extended southward from the South Georgia shelf break, and then crossing the main A23 section itself. These values had greatly reduced salinity and *δ*^18^O values, around 33.2 and –0.6‰, respectively. When the three-endmember mass balance was applied to these data it yielded meteoric water values up to 4–4.5%, around 2 percentage points higher than locations very nearby that were seemingly unaffected by glacial melt from the iceberg. The column inventories of meteoric water were also very elevated, being approximately 2.5 to 3 m; again, these showed strong contrasts to much lower values at stations in very close proximity (figure 11*d*).

It seems clear that our *δ*^18^O sampling around A68 subsampled a significantly complex field of glacial meltwater. The strong gradients in salinity, *δ*^18^O and meteoric water (figure 11) point to marked spatial structure on very small scales, which the positioning and timing of the stations were not able to fully resolve. Presumably this relates strongly to the locations of glacial melt entering the ocean itself, with multiple fragments of iceberg acting as different point sources, each melting at different rates depending on their geometries, keel depths and so on. It also likely relates to particular complexity in the patterns of ocean circulation, with the meltwater from the iceberg fragments entering an ocean region with marked mesoscale and submesoscale variability [41,42]. The injection of large amounts of meltwater likely strengthens lateral density gradients and could contribute to instabilities that enhance these structures further. Nonetheless, a general pattern emerges, with comparatively low glacial meltwater detected to the south and west of the major remnant of A68, but then higher values to the north and east from here, following an approximate trajectory of the Southern ACC Front as it loops anticyclonically around the South Georgia shelf. This would suggest the meltwater is carried ahead of the iceberg fragments themselves, though some small fragments had crossed the A23 line by this time (figure 11).

Giant icebergs like A68 are rare; smaller (though often still very large) icebergs are far more common in this vicinity [43]. Although there is great variability, icebergs generally exit the Weddell Sea and move to the east and north (as did A68), in what is colloquially termed ‘iceberg alley’ (figure 10). Since these icebergs melt as they move, they will inevitably inject glacial meltwater to the ocean, some of which will cross the A23 section. Our data show no other cases as profound as A68 in this context, though there is an apparent elevation of meteoric water in the vicinity of the South Scotia Ridge in early 2018 (figure 9*c*), with values raised to approximately 3%. Comparison with the trajectories of icebergs around this time (figure 10) shows that a large cluster of icebergs crossed the central part of the western Weddell Sea in the preceding year and then exited the Weddell Sea across the South Scotia Ridge. While meltwater from any or all of these icebergs could influence the A23 section, one of the icebergs (designated A61; green in figure 10) crossed A23 itself. While this does not constitute proof, we speculate that the elevated meteoric water concentration seen on the South Scotia Ridge in early 2018 could be due to meltwater from this cluster of icebergs.

## 6. Summary and Conclusion

The two events that are the central foci of this paper are remarkable in their magnitudes. The decline in Antarctic sea ice during 2016 is unprecedented in the satellite era, with rates of retreat dwarfing those seen even in the Arctic [18]. The giant A68 iceberg was the fourth largest iceberg ever tracked in satellite observations and the largest since B15A in 2002 [39]. It is inevitable that each will have marked impacts on the oceanography of the regions that they influence, though it is not straightforward to unequivocally determine those impacts when contemporaneous forcings from multiple other sources exist. Changes to the upper-ocean freshwater balance are clearly implicated, but salinity measurements alone are inadequate in disentangling the injection and removal of freshwater by different processes. We have demonstrated here that the use of an additional ocean tracer (*δ*^18^O), with the extra degree of freedom that it provides, enables quantitative insight into the relative importance of the changing freshwater sources.

We find that, in response to the rapid retreat of sea ice in the Weddell Sea following its record maximum in 2015, a large pulse of freshwater from sea ice melt passed through the area. A small temporal lag exists between the retreat of the solid sea ice and the peak sea ice melt contribution in the northern Weddell Sea, which we ascribe to the advective timescale for the affected waters to reach the sampling site, combined with a timescale of accumulation of sea ice melt in the ocean. We also note that our quasi-annual temporal sampling is sparse in this context.

While the absolute values of the sea ice melt contribution presented here are sensitive to choices of endmembers, changes between times or locations are much less influenced. Nonetheless, it is interesting that some initial sea ice melt percentages in the northern Weddell Sea (before the freshwater pulse moved through) were negative, given that this locality is an area of predominant net sea ice melt. We tested the possibility of seasonal aliasing of the cruise data being a factor; while all the cruises were conducted in the austral summer, there were inevitable differences in the actual months of occupation of the sections. However, we found this to be negligible in terms of impact on the sea ice melt signal observed. It seems likely that the negative sea ice melt percentages, denoting net sea ice formation from the waters sampled, were a reflection of strong sea ice production prior to the record-length maximum in 2015, again combined with advection from the major ice production regions further south.

**Figure 12.**
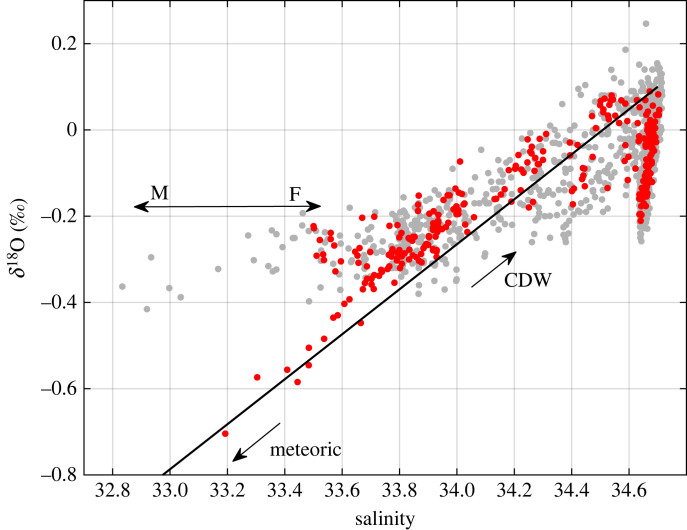
Salinity versus *δ*^18^O for samples collected on JC211 (red), including targeted samples collected in the vicinity of the A68 iceberg. The grey envelope shows all other samples from the datasets used here. (Online version in colour.)

In contrast with the sea ice melt anomalies, which covered a very broad area of the northern Weddell Sea, the melt anomalies from the giant A68 iceberg were much more localized. While the iceberg discharged a considerable quantity of freshwater to the ocean (802 ± 34 Gt of ice was lost from A68a in 3.5 y [9]), this still represents a comparatively small flux given the spatial scales over which the freshwater is distributed. If salinity measurements alone were available, it would not have been straightforward to trace the meltwater from the iceberg: the salinities of the waters were largely (though not entirely) encompassed within the typical range of salinities at this location (figure 12). Conversely, the meltwater sampled was uniquely isotopically light for this region, emphasizing the usefulness of the tracer (figure 12). While the iceberg itself disintegrated upon reaching the vicinity of the southern and western South Georgia shelf, the freshwater appears to be drawn anticyclonically around the island to its northern side. It has been seen previously that anomalous horizontal gradients in salinity and density around the South Georgia shelf can drive shelf break currents, the strength and even direction of which are sensitive to interannual changes in forcing [41,44]. This can have consequences for shelf-open ocean exchange, and the fate of biologically-relevant substances such as sedimentary- and glacially-derived micronutrients [45]. Ongoing work is exploring these aspects of the impact of A68.

The example of A68, being a marked source of isotopically-light waters locally to South Georgia, highlights the role that large icebergs can play in modulating the freshwater balance in ecologically-sensitive regions. The trajectory of A68 is not atypical for an iceberg exiting the Weddell Sea through ‘iceberg alley’, though a range of other trajectories both west and south of South Georgia also exists (figure 10). Those that follow the latter route will inevitably cross our A23 sampling section. Though tracing the impact on the ocean of smaller icebergs is even more challenging than it was for A68, we have observed that a cluster of icebergs that left the Weddell Sea around 2017/18, with some following this route, coincided with a discernible increase in meteoric water around the South Scotia Ridge in 2018. While it is not possible to definitively associate these two observations, their contemporaneity seems at least suggestive of a link. An alternative hypothesis would be that the elevated meteoric water around the South Scotia Ridge in early 2018 was due to snow accumulating on top of the sea ice, and subsequently melting into the ocean simultaneously with the strong sea ice melt observed that year. This may be a contributory factor; however, it should be noted that the elevated meteoric water seen in early 2018 was not observed in 2017 even though the sea ice melt had already started increasing then, thus it seems unlikely that this was a major causal influence.

There is an interesting possible linkage of two of the anomalous freshwater patterns observed here, with the exit of icebergs from the Weddell Sea in 2017–2018 likely to have been facilitated at least partially by the corresponding dramatic sea ice retreat. It is known that icebergs close to Antarctica can be held comparatively immobile in the ice pack for extended periods, thus it is feasible that a thinner and less extensive sea ice field permitted greater iceberg mobility. This is speculative but, if confirmed, would constitute a striking connection between meteoric and non-meteoric freshwater inputs to the ocean, which our oxygen isotope tracer is capable of decoupling.

Each of the signals reported on here will inevitably change in future. Sea ice is expected to retreat in a warming world, and projections based on climate and Earth System Models generally depict strong reductions in Antarctic sea ice by the end of this century [46,47]. However, these same models typically predict that Antarctic sea ice should have been retreating for the past several decades, a period during which it advanced significantly, if only slowly. The recent unexpected rapid retreat of sea ice, from a record-length high to a new minimum low, emphasizes that Antarctic sea ice variability is not well captured in these models, and our predictive skill concerning its future evolution is limited. Nonetheless, to the extent that strengthening external forcing will sustain a long-term diminution in sea ice extent, our expectation is that a new background level for sea ice melt in the ocean will persist, but that any further large shifts in sea ice will show a similar multi-year pulse response as per our observations. It should be noted, however, that the 2016 sea ice retreat was unprecedented in its magnitude, and further pulses could well be more subtle in form and magnitude. The observation of a new record minimum sea ice extent in February 2022 motivates ongoing research into ocean impacts; we have collected isotope samples across the Weddell Sea during this period, and these will be analysed and interpreted in the context of the variability and changes seen here.

Projections of glacial ice discharge from Antarctica appear more robust than projections of sea ice changes: it is known with higher confidence that the ice shelves, ice sheets and glaciers will contribute increasing amounts of freshwater to the Southern Ocean over the coming decades, though the absolute amounts will depend strongly on factors such as our success in limiting greenhouse gas emissions [46,47]. This enhanced discharge of glacial melt is likely to raise the general background levels of meteoric water prevalence in regions such as the Weddell and Scotia Seas. Increased iceberg calving will contribute further to this signal and likely introduce more extremes of variability via increased point-source freshwater injection to the ocean at locations distal from Antarctica itself. Such changes, and the potential future sea ice melt changes discussed above, will have consequences for upper-ocean stratification, primary production, heat and carbon exchanges between the atmosphere and the ocean interior, and ultimately global climate. Our ongoing isotope sampling in these key regions will generate further knowledge of the mechanisms and dependencies by which these influences are exerted.

## Data Availability

HADisst data are available at https://www.metoffice.gov.uk/hadobs/hadisst/. Brigham Young University iceberg tracking data are available at https://www.scp.byu.edu/data/iceberg/database1.html. Iceberg outlines were traced from satellite images available from https://wvs.earthdata.nasa.gov/. ACE isotope data are available at https://zenodo.org/badge/DOI/10.5281/zenodo.1494915.svg [27]. ACE salinity data are available at https://zenodo.org/record/1494924 [28]. ORCHESTRA/ENCORE cruise data are available via the British Oceanographic Data Centre at: https://www.bodc.ac.uk/data/bodc_database/nodb/cruise/16042/ (JR15006). https://www.bodc.ac.uk/data/bodc_database/nodb/cruise/16298/ (JR16004). https://www.bodc.ac.uk/data/bodc_database/nodb/cruise/16403/ (JR17003). https://www.bodc.ac.uk/data/documents/cruise/17517/#pd (DY113). https://www.bodc.ac.uk/data/bodc_database/nodb/cruise/17790/ (JC211). DOIs for the ORCHESTRA/ENCORE cruise datasets are: JR15006: https://www.bodc.ac.uk/data/published_data_library/catalogue/10.5285/e6fe4a24-5479-14b1-e053-6c86abc0223f/. JR16004: https://www.bodc.ac.uk/data/published_data_library/catalogue/10.5285/e0c61cc9-aa60-1e84-e053-6c86abc089b9/. JR17003: https://www.bodc.ac.uk/data/published_data_library/catalogue/10.5285/e4c8d396-13d0-3186-e053-6c86abc09138/. DY113: https://www.bodc.ac.uk/data/published_data_library/catalogue/10.5285/e7efa26d-1fda-26e8-e053-6c86abc074c3/. JC211: https://www.bodc.ac.uk/data/published_data_library/catalogue/10.5285/e4e45cd9-f1c1-5b7a-e053-6c86abc0c363.
